# Prospective clinical cohort study on the accuracy of magnetic resonance imaging in preoperative staging for cancer of the rectum with node-by-node analysis by pathologists

**DOI:** 10.1590/1806-9282.20250301

**Published:** 2025-07-07

**Authors:** Lucia Ihnát Rudinská, Ilker Sengul, Jozef Škarda, Demet Sengul, Anton Pelikán, Pavel Koscielnik, Peter Ihnát

**Affiliations:** 1 University Hospital Ostrava, Department of Forensic Medicine – Ostrava, Czech Republic.; 2 Giresun University, Faculty of Medicine, Division of Endocrine Surgery – Giresun, Turkey.; 3 Giresun University, Faculty of Medicine, Department of General Surgery – Giresun, Turkey.; 4 University of Ostrava, Institute of Molecular and Clinical Pathology and Medical Genetics, Faculty of Medicine – Ostrava, Czech Republic.; 5 Giresun University, Faculty of Medicine, Department of Pathology – Giresun, Turkey.; 6 University of Ostrava, Faculty of Medicine, Department of Surgery – Ostrava, Czech Republic.; 7 Tomas Bata University in Zlin, Faculty of Humanities – Zlin, Czech Republic.; 8 University Hospital Ostrava, Department of Radiology – Ostrava 708 52, Czech Republic.; 9 University Hospital Ostrava, Department of Surgery – Ostrava, Czech Republic.; 10 University of Ostrava, Faculty of Medicine, Department of Surgical Studies – Ostrava, Czech Republic.

**Keywords:** Magnetic resonance imaging, Rectal cancer, Lymph nodes, Lymphadenopathy, Accuracy

## Abstract

**OBJECTIVE:**

Multidisciplinary treatment is the standard of care for patients with cancer of the rectum. Neoadjuvant therapy decisions rely on the local carcinoma stage's magnetic resonance imaging assessment. However, the reliability of magnetic resonance imaging in evaluating mesorectal lymphadenopathy remains highly questionable.

**METHODS:**

A prospective clinical study was conducted to assess the accuracy of preoperative magnetic resonance imaging in assessing T-stage and N-stage rectal carcinoma. The magnetic resonance imaging findings were compared with those of a detailed histopathological examination of rectal specimens in each patient.

**RESULTS:**

The study included 48 patients with cancer of the rectum who had undergone rectal resection with total mesorectal excision. The magnetic resonance imaging assessment of the T-stage was accurate in 26 out of the 48 cases (54.2%). Overstaging of the T-stage was recognized in 18 patients (37.5%), and 4 (8.3%) were understaged. According to preoperative magnetic resonance imaging (using Horvat's criteria), 56 suspicious/malignant lymph nodes were identified. However, only 13 (23.2%) of these lymph nodes were proved malignant on histopathology examination. The sensitivity of magnetic resonance imaging for nodal involvement assessment was 52.0% and specificity was 93.6%. Of the 646 magnetic resonance imaging-negative lymph nodes, 12 (1.9%) were false negatives (histopathology revealed metastatic lymph nodes). Of note, 20 study patients with malignant lymphadenopathy (according to magnetic resonance imaging) were detected. The magnetic resonance imaging diagnosis of malignant lymphadenopathy was correct in only 30.0% of these patients.

**CONCLUSION:**

The accuracy of magnetic resonance imaging in evaluating mesorectal lymph nodes for cases with cancer of the rectum is remarkably low. Therefore, neoadjuvant therapy indications should be based primarily on the magnetic resonance imaging description of the depth of carcinoma invasion.

## INTRODUCTION

Multimodal treatment (surgery, radiotherapy, and chemotherapy), *per se*, represents the current standard of treatment management for patients diagnosed with rectal cancer. The selection of the primary treatment modality is predominantly based on the locoregional status of the disease^1,2^. Patients with locally advanced rectal cancer, as determined on imaging (clinical stages T3, T4, or N+), are recommended to undergo neoadjuvant chemoradiotherapy (CRT) followed by surgery. Nevertheless, the indications for neoadjuvant treatment remain controversial^3-6^.

In fact, magnetic resonance imaging (MRI) has emerged as the most precise modality for assessing the locoregional status of rectal cancer^2-7^, which serves two main purposes: assessing the primary tumor (T stage) and evaluating nodal involvement. MRI exhibits a high level of accuracy in determining the depth of tumor invasion^8-10^, but the reliability of MRI assessment of nodal involvement remains quite questionable. Relying solely on size criteria for MRI nodal assessment often leads to false-positive diagnoses. Furthermore, most metastatic lymph nodes (LNs) measure <5 mm^11,12^. The morphological characteristics of LNs on MRI have shown the potential to predict nodal status more effectively than size alone^11^. A combined evaluation of nodal size and morphological criteria has been proposed to address this. However, suspicious LNs are defined as (i) nodes >9 mm; (ii) nodes sized 5–9 mm meeting at least two of three criteria (round shape, irregular borders, and heterogeneous signal intensity); and (iii) nodes <5 mm meeting all the three morphological criteria^13^.

Despite the importance of accurate MRI assessment of rectal cancer, only a limited number of studies have meticulously matched T-stage and individual LNs visualized on MRI, with their histological counterparts following total mesorectal excision (TME)^11,14,15^. To the best of our knowledge, this is the first study examining the exactness of the novel MRI criteria (size+morphological criteria) for nodal assessment. Therefore, our study's objective is to evaluate the accuracy of preoperative MRI in assessing T-stage and LN involvement (using the novel criteria) on a node-by-node basis in rectal cancer cases.

## METHODS

### Study design

This prospective clinical cohort study was conducted at University Hospital Ostrava in the Czech Republic and received approval from the hospital's Ethics Committee (ref. number 371/2021). All the cases that underwent surgical resection of rectal cancer within the study period (January 1, 2021–June 30, 2023) were considered for eligibility. Written informed consent was obtained from all the enrolled patients, and measures were taken to ensure their anonymity.

The main objective of this study was to assess the exactness of preoperative MRI in determining the T-stage and nodal status of rectal cancer patients. This was achieved by confronting the MRI findings with the histopathology reports node by node. Patients undergoing neoadjuvant treatment were not included in the study. Pelvic irradiation may downstage a rectal tumor and change an originally malignant LN into a benign one or a fibrotic scar; therefore, comparing MRI findings with histopathological findings would not be objective in irradiated patients.

The inclusion criteria for the study were as follows: age ≥18 years, having rectal cancer, and underwent an elective rectal resection with TME, while the exclusion criteria were the cases if they had generalized or recurrent disease, underwent neoadjuvant treatment, were unable to undergo radical surgical resection, or refused to participate.

### Magnetic resonance imaging assessment of nodal involvement

All the studied cases had undergone pelvic MRI within 2 weeks prior to surgery, using a 3T system (Magnetom Prisma 3T, XA 30, Erlangen, Germany). A standard MRI protocol was employed, which comprised pre-contrast sagittal T2-weighted (T2W) images, followed by obtaining axial and oblique coronal T2W images. The imaging parameters were as follows: repetition time: 2,120–2,680 ms using a restore pulse; echo time: 96 ms; 3 mm slice thickness; a field of view of 200 mm for coronal, sagittal, and axial views; and an image matrix of 512×448.

A radiologist with a minimum of 10 years of experience in MRI assessment evaluated the MRI images. The radiologist reviewed the MRI independently without referring to computed tomography or endorectal ultrasonography outcomes. The radiologist described the location, size, and shape of LNs identified on the scans. In contrast, the location of all suspicious LNs (MRI malignant) was marked on a diagram and provided to the pathologist.

The MRI criteria distinguishing non-metastatic from metastatic LNs comprised size, margin, and T2W signal intensity characteristics^11,13^. A metastatic LN was defined as a node >9 mm, a node measuring 5–9 mm and meeting at least two of three criteria (round shape, irregular borders, and heterogeneous signal intensity), or a node <5 mm and meeting all the three morphological criteria^13^.

### Surgical procedures

All the patients were managed through multidisciplinary team meetings, following the guidelines provided by the National Comprehensive Cancer Network (NCCN), and certified surgeons with at least 10 years of experience in colorectal cancer surgery performed the surgery (rectal resection with TME). After initial laparoscopic abdominal cavity exploration, inferior mesenteric vessels were identified, clipped, and divided. Furthermore, the TME was performed in a standardized manner to achieve high-quality mesorectal excision. Depending on the location of the tumor, one of three surgical procedures was employed: (i) low anterior resection (LAR), (ii) transanal TME (TaTME), or (iii) abdominoperineal resection (APR). Following surgery, the resected specimens were promptly transported to the pathology laboratory.

### Histopathological examination

All the resected specimens were sliced transversely at 5-mm intervals. The slices were carefully matched with the corresponding MR images to enable a node-by-node comparison of histological slides with MRI scans. All the tissue blocks with a maximum size of 5.0 cm×7.0 cm×0.5 cm were cut and inserted into plastic megacassettes in order to facilitate whole-mount sections. Following fixation in 10% buffered formalin, the tissue blocks underwent processing in a Microm STP-120 tissue machine. Afterward, the processing protocol included an ascending series of alcohol (21 h), clarification in xylene (6 h), and saturation in melted paraffin (minimum 6 h). Furthermore, the sections measuring 3–5 μm from paraffin mega blocks were prepared and stained with hematoxylin-eosin. After identification, the harvested LNs from each specimen were put in individual trays numbered according to the nodes identified on the MRI scans. Finally, the histopathology of each LN was reported based on its tray number.

### Data collection and statistical analysis

All the data for this study were prospectively collected, and the demographic and clinical data were entered into the study database prior to surgery. The radiologist plotted the location of all the suspicious (MRI malignant) LNs on a scheme delivered to the pathologist. The findings from the MRI scans and histopathology reports were recorded in the study database. As such, descriptive statistics were used to analyze the collected data, and a level of α=0.05 (p<0.05) was considered statistically significant.

## RESULTS

In total, 48 patients with rectal cancer were prospectively included in the study. The mean age of the study cases was 64.6±9.1 years; the mean body mass index (BMI) was 29.3±4.3 kg/m^2^. The mean distance of the rectal tumor from the anal verge was 5.7±2.9 cm. According to the MRI staging, 13 (27.1%) study patients were classified as stage I, 12 (25.0%) as stage II, and 23 (47.9%) as stage III. The LAR was performed in 20 (41.7%) study cases, while the TaTME in 5 (10.4%) and APR in 23 (47.9%) study cases. Table 1 presents the outcomes of the MRI assessment of the T-stage of rectal carcinoma.

**Table 1:** T-stage of rectal tumors (magnetic resonance imaging assessment of clinical T-stage and histopathologic classification).

	Histopathologic T-stage (pT) (n=48)			
	Adenoma	T1	T2	T3
**Clinical T-stage (cT)**				
T1	0	2	0	0
T2	0	2	10	4
T3	3	3	10	14

The histopathological examination revealed adenoma in 3 (6.2%) patients, T1 tumor in 7 (14.6%), T2 tumor in 20 (41.7%), and T3 tumor in 18 (37.5%). In addition, the overall accuracy of MRI in determining the T-stage was 54.2% for all the cases. The sensitivity of MRI was 28.6% for T1 tumors, while 50.0% for T2 and 77.8% for T3. Moreover, the specificity of MRI was 100.0% for T1 tumors, 82.3% for T2 and 65.2% for T3. Regarding the T-stage, overstaging was noted in 18 (37.5%) patients, while 4 (8.3%) were understaged. A sum of 702 LNs was examined histopathologically, and the mean number of LNs per specimen was 14.6±4.2. Table 2 exhibits data regarding the accuracy of MRI in assessing mesorectal lymphadenopathy.

**Table 2:** Magnetic resonance imaging and histopathological assessment of mesorectal lymph node positivity/negativity.

		Number of lymph nodes n (%) (n=702)
MRI-positive (suspicious) LNs		56 (7.9)
	Histopathology malignant	13 (1.8)
	Histopathology benign	43 (6.1)
MRI-negative (non-suspicious) LNs		646 (92.0)
	Histopathology benign	634 (90.3)
	Histopathology malignant	12 (1.7)

MRI: magnetic resonance imaging; LNs: lymph nodes.

Of the 646 LNs that were MRI negative (not visible on MRI or not fulfilling the criteria for malignancy), 634 (98.1%) were histologically confirmed as genuinely negative. In contrast, 12 (1.9%) were false negatives (histopathology revealed metastatic LNs). The MRI sensitivity for nodal assessment was 52.0% and the specificity was 93.6%.

Although the success rate of matching MRI-negative LNs was 98.1%, the MRI's exactness in predicting positive LNs was very low in the study. Of the 56 LNs considered malignant on preoperative MRI scans, only 13 (23.2%) were histologically confirmed as truly malignant, while 43 (76.8%) were not on histology examination. The study's success rate of matching MRI-positive LNs was 23.2% (13 malignant LNs out of 56 MRI-positive LNs). The nodal assessment on preoperative MRI in individual studied cases is presented in Table 3. A total of 20 patients had malignant lymphadenopathy according to preoperative MRI, and the exactness of MRI-positive nodal assessment was only 30.0% (histologically malignant LNs were found in 6 of the 20 patients). The determination of negative LNs on preoperative MRI was correct in 89.3% of our patients (histopathology confirmed negative LNs in 25 of 28 patients preoperatively classified as cN0). On top of that, malignant LNs were proved in 10.7% of our patients classified initially as cN0.

**Table 3:** Magnetic resonance imaging and histopathological assessment of nodal status in individually studied patients.

		Number of patients n (%) (n=48)
Patients with MRI-positive (suspicious) LNs		20 (41.7)
	pN0 (pathological stage N0)	14 (29.2)
	pN+ (pathological stage N+)	6 (12.5)
Patients with MRI-negative (non-suspicious) LNs		28 (58.3)
	pN0 (pathological stage N0)	25 (52.1)
	pN+ (pathological stage N+)	3 (6.2)

MRI: magnetic resonance imaging; LNs: lymph nodes.

## DISCUSSION

In managing cases with rectal carcinoma, MRI is considered the most accurate imaging modality for evaluating the locoregional status of carcinoma of the rectum^7,8,16^. The principal goal of pelvic MRI is to determine whether neoadjuvant treatment should be applied or surgery can be performed as the primary treatment modality. Neoadjuvant radiochemotherapy is typically recommended for patients with locally advanced carcinoma (cT3–T4) and/or patients with locoregional lymphadenopathy (cN+).

According to the available literature, MRI allows the evaluation of the depth of tumor invasion with satisfactory precision^8-10^. Brown et al.^17^ reported excellent accuracy in assessing the extramural invasion of T3 tumors; the tumor stage was correctly diagnosed in all 25 consecutive cases. However, the difference between the depth of extramural invasion measured on MRI and the corresponding measurements on histopathology slices ranged from −5.0 mm to +5.5 mm. We believe 5 mm is a large deviation when differentiating between the T-stages of rectal cancer. They concluded that MRI provides valuable information for identifying T3 tumors for neoadjuvant therapy in patients who are at a high risk of failure with complete excision^17^. Beets et al.^10^ reported a substantially lower exactness of preoperative MRI in assessing tumor T-stage (a study group of 76 patients). The histopathology stage agreed with the MRI tumor stage in 83% of the cases of the first observer and 67% for the second^10^. Similar outcomes were published by Blomqvist et al.^18^ MRI tumor stage correlated with histopathology stage in 32 of 49 patients (65.3%). They emphasized that the tumor penetration of the rectal wall was predicted to possess 86% sensitivity and 65% specificity.

Park et al.^15^ reported an overall T-stage accuracy of 83% (a study group of 40 patients). The T-stage diagnostic accuracy was 69% in the subgroup of patients after neoadjuvant radiotherapy (RT) and 89% in the subset of patients without neoadjuvant treatment. The authors noted overstaging in 15% and understaging in 3% of the study patients.

In our study, the overall T-stage accuracy of MRI was 54.2%; we overstaged more than one-third of our patients (37.5%). In addition to the low accuracy of MRI, three patients with an MRI diagnosis of T3 carcinoma were classified as large adenomas by histopathology (see Table 2).

In the available literature, only a few studies investigate the exactness of the MRI assessment of nodal status by comparing individual LNs seen on MRI with their histopathology examination^11,14,15^. Brown et al.^17^ published a study involving 42 patients with 437 harvested LNs. The authors reported a very high MRI accuracy (sensitivity 85% and specificity 97%) if a node was considered suspicious due to an irregular border or mixed signal intensity.

Park et al.^15^ published a significantly lower accuracy of MRI in nodal assessment (sensitivity 58% and specificity 88.4%). A sum of 120 LNs <3 mm were not identified on MRI, and 18 of these small LNs (15%) were found malignant on histopathology. They stated that MRI's exactness was poor for malignant LN detection, as evidenced by the low positive detection rate and sensitivity. Additionally, Guillem et al.^14^ also reported the low exactness of nodal assessment on MRI (a study group of 188 patients). All the study patients were staged as T3 N0 using MRI and endorectal ultrasound, but histopathological examination revealed malignant LNs in 22% of these patients despite neoadjuvant CRT. They proclaimed that the exactness of MRI and endorectal ultrasound in detecting malignant mesorectal LNs is very limited.

Consistent with these findings, we have also demonstrated the very poor exactness of MRI in predicting the nodal status of patients with rectal cancer. Only 23.2% of MRI-suspicious LNs were found malignant on histopathology examination. Among the 20 patients with MRI-suspicious LNs, only 30.0% had histopathologically confirmed malignant LNs. In other words, if a positive LN was seen on MRI scans, there was only a 30.0% chance of being correct.

The main strengths of our study lie in its concept/design, which evaluated the precision of the novel MRI criteria, as well as the meticulous node-by-node analysis conducted by pathologists. A limited number of included patients and a single-center study design present the main study limitations.

## CONCLUSION

The outcomes of our study indicate that MRI preoperative staging of rectal cancer, especially the assessment of mesorectal LN involvement, is inaccurate and unreliable. Not even implementing the novel criteria for MRI nodal assessment is associated with satisfactory outcomes. Therefore, neoadjuvant CRT should not be indicated only based on malignant lymphadenopathy seen on MRI scans. As such, only the depth of tumor invasion should be a justifiable reason for initiating neoadjuvant treatment.

## Data Availability

The datasets generated and/or analyzed during the current study are available from the corresponding author upon reasonable request.
